# A Novel Application of B.EL.D™ Technology: Biosensor-Based Detection of *Salmonella* spp. in Food †

**DOI:** 10.3390/bios14120582

**Published:** 2024-11-29

**Authors:** Lazaros Konstantinou, Eleni Varda, Theofylaktos Apostolou, Konstantinos Loizou, Lazaros Dougiakis, Antonios Inglezakis, Agni Hadjilouka

**Affiliations:** 1 EMBIO Diagnostics Ltd., Athalassas, 2018 Nicosia, Cyprus; lazaros.konstantinou@embiodiagnostics.eu (L.K.); e.varda@embiodiagnostics.eu (E.V.); theo.apo@embiodiagnostics.eu (T.A.); k.loizou@embiodiagnostics.eu (K.L.); l.dougiakis@embiodiagnostics.eu (L.D.); a.inglezakis@embiodiagnostics.eu (A.I.); 2 Department of Life Sciences, School of Sciences, European University of Cyprus, 2404 Nicosia, Cyprus

**Keywords:** *Salmonella*, cell-based biosensor, bioelectric recognition assay, membrane-engineering, food, meat, meat products

## Abstract

The prevalence of foodborne diseases is continuously increasing, causing numerous hospitalizations and deaths, as well as money loss in the agri-food sector and food supply chain worldwide. The standard analyses currently used for bacteria detection have significant limitations with the most important being their long procedural time that can be crucial for foodborne outbreaks. In this study, a biosensor system able to perform robust and accurate detection of *Salmonella* spp. in meat products was developed. To achieve this, a portable device developed by EMBIO Diagnostics called B.EL.D^TM^ (Bio Electric Diagnostics) and cell-based biosensor technology (BERA) were used. Results indicated that the new method could detect the pathogen within 24 h after a 3-min analysis and discriminate samples with and without *Salmonella* with high accuracy. Achieving an accuracy of 86.1% and a detection limit (LOD) of 1 log CFU g^−1^, this innovative technology enables rapid and sensitive identification of *Salmonella* spp. in meat and meat products, making it an excellent tool for pathogen screening.

## 1. Introduction

Salmonellosis, an infection caused by *Salmonella* bacteria, is one of the greatest global public health issues [1]. With 93.8 million cases of gastroenteritis and approximately 155,000 deaths annually, non-typhoidal *Salmonella* is one of the four major causes of diarrheal disorders worldwide. It is also a prevalent cause of human bacterial enteritis [2]. A member of the *Enterobacteriaceae* family, *Salmonella* spp. are rod-shaped, gram-negative bacteria. There are currently around 2500 distinct serotypes or serovars among *Salmonella bongori* and *Salmonella enterica*, the two species of *Salmonella* [3]. *Salmonella enterica* ser. Enteritidis and *Salmonella enterica* ser. Typhimurium (Nontyphoidal *Salmonella*, N.T.S.) are two of the most significant *Salmonella* serotypes that are transmitted from animals to humans [4,5]. The first one is the most frequently reported serovar in human salmonellosis cases in the EU and the United States, and the latter one occupies the second position in the EU [6] and third in the United States [7] in human salmonellosis reports, while it represents the most prevalent and disseminated serovar globally [8].

*Salmonella* spp. can survive for several weeks in dry environments and for several months in water, while it can overcome numerous barriers and adapt to different conditions, such as high temperatures and low pH [9]. Due to its ubiquitous nature, it can contaminate people when they consume raw or undercooked food and when food handlers have inadequate cleanliness during preparation processes [10]. Furthermore, due to its adaptability, it can survive in the intestines of humans and animals and, therefore, is isolated from patients’ stools. Additionally, there is a possibility that pets could contract the disease after eating tainted food and can be transmitted to people who come in touch with them [11]. Salmonellosis-causing foods can be classified into four categories: (i) raw or undercooked eggs, (ii) unpasteurized dairy products, (iii) fruits and vegetables, and (iv) red meat, poultry, and shellfish [12]. Since *Salmonella* was identified as a foodborne pathogen, measures were developed to control its incidence in food processing plants and to reduce the risk of its transmission [1]. Nevertheless, salmonellosis infections are constantly being reported resulting in a significant number of hospitalizations and deaths.

The infectious dose of the pathogen can vary greatly, as it depends upon the species, the strain, the mode of transmission, the host’s immune response, and the food substrate. According to studies on reported outbreaks, the infectious dose is usually very low—less than 1000 cells [13]. Hence, the Commission for food safety regulation (EC) N◦ 2073/2005 has established ‘zero tolerance’ policy in a specified amount of a given food product [14]. The same policy has been established by the Food and Drug Administrator (FDA) and the U.S. Department of Agriculture (USDA) for *Salmonella* spp. in certain foods [15]. This highlights the risk that arises from the presence of *Salmonella* in foodstuff and the necessity for the pathogen’s detection and proper control. The current methods for detecting *Salmonella* spp. in food products involve traditional culture methods regarded as the gold standard, combined with molecular and immunological approaches. More accurately, according to the ISO 6579-1:2017 standard [16], pathogen detection involves four main steps: (i) pre-enrichment in a non-selective broth to revive any stressed bacteria, (ii) selective enrichment in broths that promote *Salmonella* growth, (iii) plating samples on selective agar media, and (iv) conducting biochemical and serological tests on suspected colonies for confirmation. In addition, molecular-based techniques such as the Polymerase Chain Reaction (PCR), offer faster and highly sensitive detection in food matrices. Although the present detecting techniques may be quite effective, they have several drawbacks due to their lengthy procedure times (culture-based techniques), high costs, need for highly skilled personnel (molecular-based techniques), and limited detection sensitivity (immunology-based methods) [17]. The most recent methods for detecting *Salmonella* in food products [17,18,19] along with a comparison of their advantages and disadvantages, are presented in Table 1:

With multiple studies reporting excellent accuracy and sensitivity in an expanding area of applications (e.g., food quality and safety control, environmental monitoring, clinical diagnostics), novel methodologies, such as the biosensing techniques, have been the focus of scientific interest [20]. Studies have noted the development of quick and accurate immune-based sensors to detect the presence of *Salmonella* spp. in food, including piezoelectric biosensors [21], optical immunosensors based on titanium dioxide nanoparticles [22], and electrochemical biosensors [23]. Nevertheless, even though live cell-based biosensor systems have been already employed in several research with great effectiveness, very few studies have been undertaken utilizing cell-based biosensor for detecting *Salmonella* spp. in food and environmental samples [24].

The purpose of this study was to develop and validate a new biosensor system for the detection of *Salmonella* spp. in food. The newly developed system utilizes two primary axes: (i) a cell-based biosensor technology that gauges changes in cell membrane potential as determined by the Bioelectric Recognition Assay (BERA) principle and (ii) a portable device created by EMBIO Diagnostics called B.EL.D^TM^ (Bio Electric Diagnostics). An Android or iOS device can connect to the system through Bluetooth 4.0, enabling the end-user to receive instant notification of the test result. Since salmonellosis is mainly linked to poultry, meat, and egg consumption, the validation tests were performed on cured meat samples and frozen ready-to-eat meat and meat products, including burgers, sausages, turkey, and chicken fillets. Finally, when foodborne pathogens are present in food they are usually in low numbers and frequently coexist with larger populations of other microorganisms. Hence, seven protocols with different broths and incubation times were evaluated for their ability to detect and discriminate *Salmonella* spp. among other bacteria, and the ISO 6579-1: 2017 standard [16] was used as the gold standard method to validate these results.

## 2. Materials and Methods

### 2.1. Collection of Samples

Firstly (phase 1), samples (*n* = 50) of cured meat and frozen ready-to-eat meat and meat preparations were collected from a local meat processing company to develop the biosensor system and validate its ability to detect the pathogen in these food substrates. A second sampling (phase 2) (*n* = 50) was then carried out to evaluate the possibility of reducing the incubation time and the limit of detection of the pathogen as much as possible. All the samples were transported to the laboratory in cool boxes to maintain the cooling chain and were analyzed on the same day.

### 2.2. Cell Culture and Fabrication

Monkey African green kidney (Vero) cell cultures (LGC Promochem, Teddington, UK) were cultured according to Apostolou et al. [25]. Briefly, cells were cultured in Dulbecco’s medium (DMEM) (Biosera, Cholet, France) with 10% fetal bovine serum (FBS) Sigma-Aldrich (Taufkirchen, Germany), 10% streptomycin/penicillin Sigma-Aldrich (Taufkirchen, Germany), and 10% L-glutamine and L-alanine (nutrient medium) Sigma-Aldrich (Taufkirchen, Germany) and incubated at 5% CO_2_ and 37 °C. Vero cells were detached from the culture vessels by adding trypsin/EDTA (10 min at 37 °C) (Biosera, Cholet, France) and membrane-engineered cells were created, based on previously described protocols [25,26]. In particular, the cell pellet was resuspended in phosphate-buffered saline (PBS) (Biomedicals, Illkrich, France) containing anti-*Salmonella* spp. antibodies (Section 2.3) and incubated on ice for 20 min. The cell-antibody mixture was then transferred into electroporation cuvettes, and electroinsertion was achieved by applying two square electric pulses at 1800 V/cm using the Eppendorf Eporator (Hamburg, Germany). Subsequently, the mixture was incubated in nutrient medium at 37 °C with 5% CO_2_ for 24 h. After incubation, the medium was removed, and the biosensors (Vero/anti-*Salmonella* cells) were mechanically detached and collected in nutrient medium within Eppendorf tubes. 

### 2.3. Biosensor Creation and Antibody Selection

Testing antibody performance across different applications is often necessary to ensure optimal sensitivity and reliability in target detection, as antibody sensitivity can be affected by several factors (e.g., sample matrices, detection method, and epitope availability). Hence, to develop an accurate biosensor system for the detection of *Salmonella* spp., the first important step was to select a proper antibody that reacts in a sensitive and specific manner in the presence of the pathogen. To achieve this, two monoclonal mouse-hosted antibodies were initially examined: (i) the *Salmonella* species antibody (ABIN3071534) (SPA biosensor) and (ii) the *Salmonella* antibody (ABIN934004) (SA biosensor), purchased by antibodies-online.com. The two antibodies were examined at three different concentrations (1, 5, and 10 μg mL^−1^). Both antibodies recognize *Salmonella* O-serogroups A, B, C1, C2, D, E1, E3, E4, F, G1, and G2 of *Salmonella enterica* subsp. *enterica* (subsp. I), responsible for approximately 99% of salmonellosis cases in humans and warm-blooded animals [27]. The tests were conducted on 150 sodium chloride (NaCl 0.85%) samples, both without *Salmonella* spp. (control) and with *Salmonella* spp. (samples) at four different concentrations (1, 2, 3 and 6 log CFU mL^−1^). *Salmonella enterica* subsp. *enterica* serovar Enteritidis (WDCM 00030 Vitroids) and *Salmonella enterica* subsp. *enterica* serovar Typhimurium (WDCM 00031 Vitroids), (Sigma-Aldrich, Taufkirchen, Germany) were used to inoculate the samples. The performance characteristics of the six different biosensors (two antibodies x three concentrations) were calculated to determine which was the most sensitive and reliable in detecting *Salmonella* spp.

### 2.4. Cross Reactivity Assessment

After the selection of the most promising antibody and the concentration with the best results (Section 2.3), tests were conducted utilizing other *Enterobacteriaceae* members to validate the biosensor’s specificity *Escherichia coli* (WDCM 00013 Vitroids), *Klebsiella aerogenes* (WDCM 00006 Vitroids), and *Citrobacter freundii* (WDCM 00175 Vitroids) (Sigma-Aldrich, Taufkirchen, Germany) were included for this assessment. A total of 120 tests were performed in NaCl 0.85% solution inoculated with these bacteria, diluted to the four final concentrations used for the creation of the biosensors (1, 2, 3 and 6 log CFU mL^−1^) (Section 2.3). The results were compared to control samples and *Salmonella* samples to ensure no cross-reactivity with the biosensor containing the selective antibody for *Salmonella* spp.

### 2.5. Bacteria Culturing and Sample Inoculation

Prior to culture, *Salmonella enterica* subsp. *enterica* serovar Enteritidis and *Salmonella enterica* subsp. *enterica* serovar Typhimurium, *Escherichia coli*, *Klebsiella aerogenes*, and *Citrobacter freundii* were stored at −20 °C in nutrient broths supplemented with 50% glycerol. Before any experimental use, the pathogens were grown twice in Brain Heart Infusion broth (Merck, Darmstadt, Germany) at 37 °C for 24 h to achieve their revival [28]. To create and evaluate the biosensor system (Section 2.3 and Section 2.4), overnight cultures of *S.* Typhimurium, *S.* Enteritidis, *E. coli*, *K. aerogenes*, and *C. freundii* (9 log CFU mL^−1^) were centrifuged (3500 rpm/10 min), washed twice with sterile saline solution (NaCl 0.85%), re-suspended in the same diluent, and serially diluted to achieve the desired final concentrations (1, 2, 3 and 6 log CFU mL^−1^). To inoculate food samples, overnight *Salmonella* spp. cultures (9 log CFU mL^−1^) were centrifuged (3500 rpm/10 min), washed twice with sterile saline solution (NaCl 0.85%), resuspended in the same diluent, and serially diluted. Subsequently, 25 g of each food substrate were placed in sterile stomacher bags and sprayed with the appropriate pathogen dilution to achieve the desired final inoculation levels (0.6, 1, and 2 log CFU g^−1^). The subsequent sample treatment was performed according to the respective protocols (Section 2.8.1).

### 2.6. B.EL.D^TM^ Device and Sample Loading

**Figure 1:**
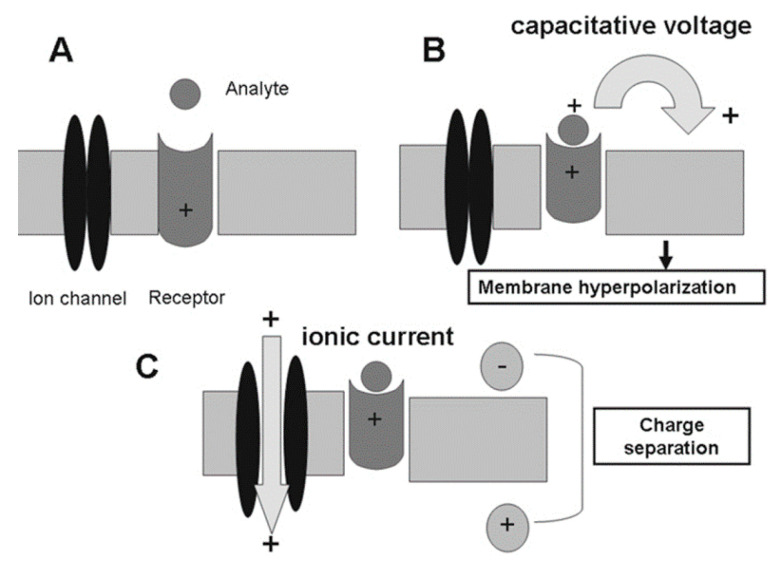
Working concept of Molecular Identification through Membrane Engineering (MIME): Initially, the cell membrane potential remains stable due to balanced ion flow through ion channels (**A**). Upon binding of the target molecule to its receptor, structural changes cause a shift in molecular charge within the membrane (**B**), leading to ion concentration on one side and membrane hyperpolarization. The opening of ion channels generates a measurable ionic current (**C**). (Reprinted from T. Apostolou, Agricultural University of Athens, 2010) [30].

The B.EL.D^TM^ device used for the development of the method and the validation of the new biosensor system is created and produced by EMBIO DIAGNOSTICS (EMBIO DIAGNOSTICS Ltd., Nicosia, Cyprus). The device, a portable multichannel potentiometer with an interchangeable connector made of eight screen-printed electrodes, measures electric signals from various elements of biorecognition. High-accuracy A/D converters are used for measurements, enabling high-throughput, and quick analysis, while the Bioelectric Recognition Assay (BERA), a potent cell-based biosensor technology, serves as the foundation for this device. Based on this technology, electroporation introduces several receptor molecules (enzymes or antibodies) into the cell membrane, improving the membrane’s capacity to identify target analytes with selectivity [29]. This method relies on measuring the shift in membrane potential that occurs when a target molecule attaches itself to the cell membrane’s embedded receptors (Figure 1). Ion flux via ion channels initially keeps the membrane potential steady (a). Once the target molecule binds to the receptor, the receptor undergoes a structural change, causing a shift in its molecular charge within the membrane (b). This leads to ion accumulation on one side of the membrane, resulting in hyperpolarization. When the ion channels open, the movement of ions generates a measurable ionic current (c) that is being measured by the device. Additionally, the device utilizes Bluetooth 4.0 to connect with an Android or iOS device, enabling the end-user to receive an immediate notification of the test results.

The analysis of the samples was conducted as previously described by Hadjilouka et al. [20,26]. In a nutshell, 20 μL of the membrane-engineered cells (~5 × 10^4^ cells) were added on each of the eight screen-printed electrodes and after 120 s, 20 μL of each sample were added on top of the membrane-engineered cells. Every measurement lasted three minutes, and for each sample, 720 data were captured at a sampling rate of four hertz (Hz). Following each study, measurements were sent to a cloud server, where they were used to instantaneously calculate results using a newly created algorithm and display them on the Android/iOS screen. Each experiment was carried out multiple times, and every sample was evaluated eight times using a set of eight different sensors.

### 2.7. Algorithm for Response Processing and Statistical Analysis

Every test generated a time series with 720 voltage detection measurements for each sample. Data analysis was conducted according to Hadjilouka et al. [26] using libraries in the python programming language. More analytically, a two-step analysis was performed, and four feature vectors were calculated based on (i) a rolling average with a window size of 50 and (ii) the average values for each data set. These vectors were calculated for each electrode channel and the entire test data set (8 electrodes). Thus, 18 feature values—8 for each channel and 1 overall value/(i) and (ii)—were used to create the algorithm and discriminate the samples. The obtained results from positive and negative samples were then compared, and a one-way analysis of variances (ANOVA) was used to determine statistical differences. The limit of detection (LOD was then established, and the thresholds that distinguish positive from negative samples were defined for each feature vector. After creating data-stored result arrays for positive and negative samples, the system was ultimately able to instantaneously categorize the samples as being above or below the LOD, after each test.

Finally, performance indicators were calculated for the new method based on the comparison of the findings acquired by the biosensor and the standard methods. These indicators were sensitivity (Se: the ability of a test to correctly classify a sample as positive), specificity (Sp: the percentage of negative samples correctly identified by the test), positive predictive value (PPV: the probability that a sample is positive given positive test result), and negative predictive value (NPV: the probability that a sample is negative given a negative test result).

### 2.8. Experimental Design

After the completion of the biosensor’s development and the cross-reactivity assessment (Section 2.3 and Section 2.4), biosensors were evaluated for their accuracy in detecting the pathogens in food samples. A total of hundred (*n* = 100) samples of cured meat and frozen ready-to-eat meat and meat preparations were tested for the development and validation of the method. From each food sample (*n* = 100), four different samples (25 g sample + 225 mL Buffered Peptone Water (BPW) (Merck, Darmstadt, Germany) were prepared for testing. Three were inoculated with *Salmonella* spp. at 0.6, 1, and 2 log CFU g^−1^ (Section 2.5) [positive samples] and one remained uninoculated to be used as the control [negative sample] after being tested negative for pathogen’s presence, according to the ISO 6579-1:2017 method [16]. The ISO method was conducted utilizing Xylose Lysine Deoxycholate agar (XLD), Müller–Kauffmann Tetrathionate (MKTTn), Rappaport–Vassiliadis–Soya (RVS), and M-broth supplied by Merck (Darmstadt, Germany). In addition, API 20E test (BioMerieux, Askim, Sweden), a standardized identification system that helps identify *Enterobacteriaceae* and other non-fastidious Gram-negative bacteria using 21 miniaturized biochemical test and database, was used for the investigation of the suspected *Salmonella* colonies.

**Figure 2:**
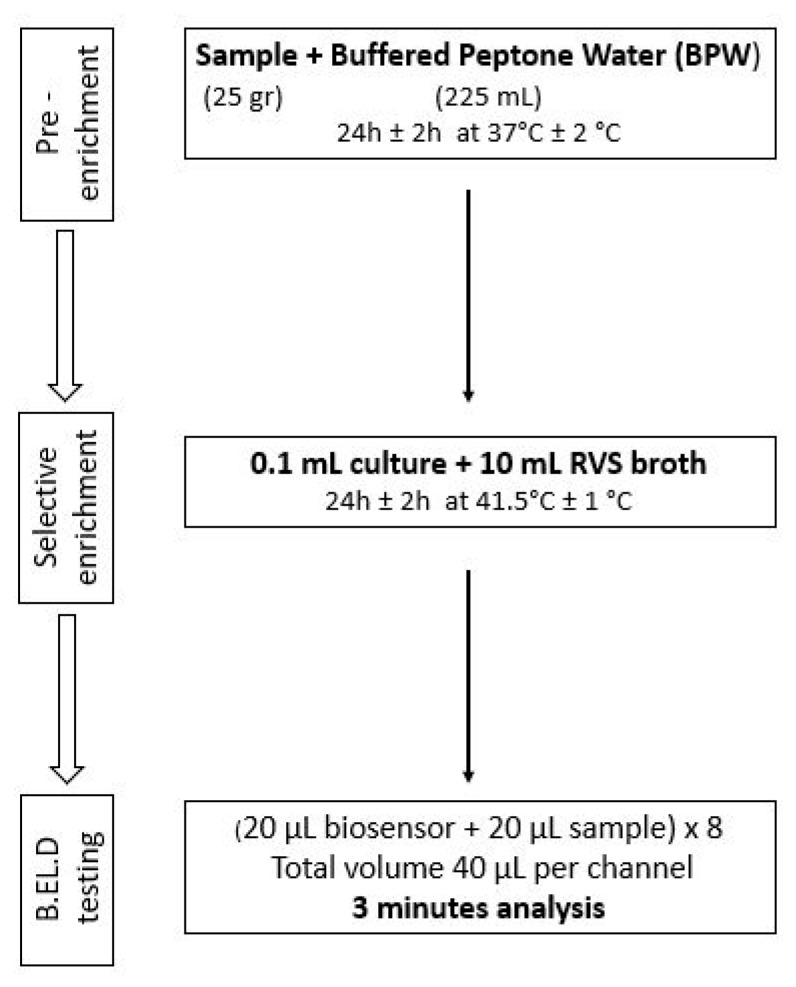
Phase 1—Protocol 1.

**Figure 3:**
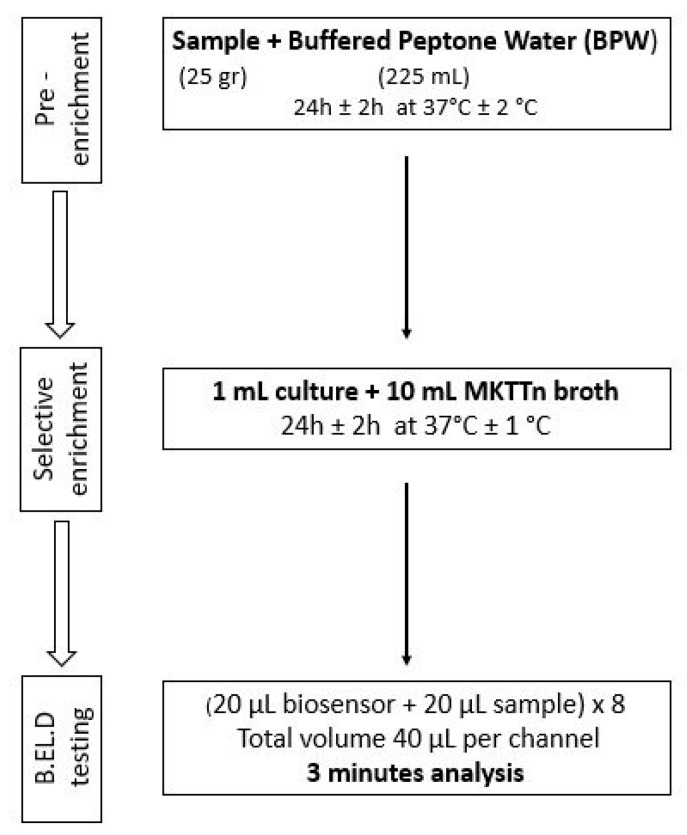
Phase 1—Protocol 2.

**Figure 4:**
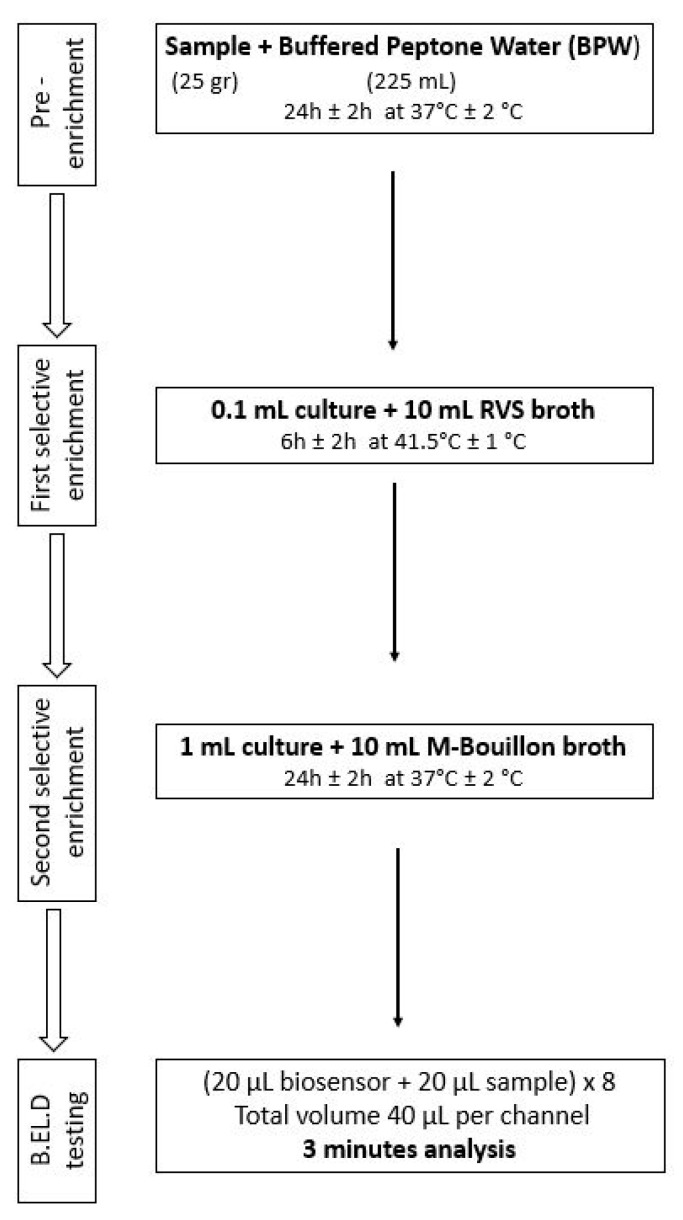
Phase 1—Protocol 3.

**Figure 5:**
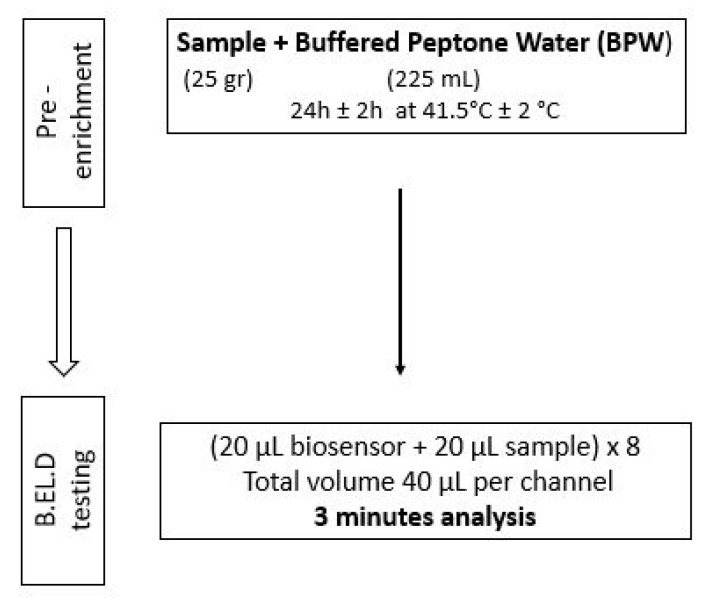
Phase 2—Protocol 4.

**Figure 6:**
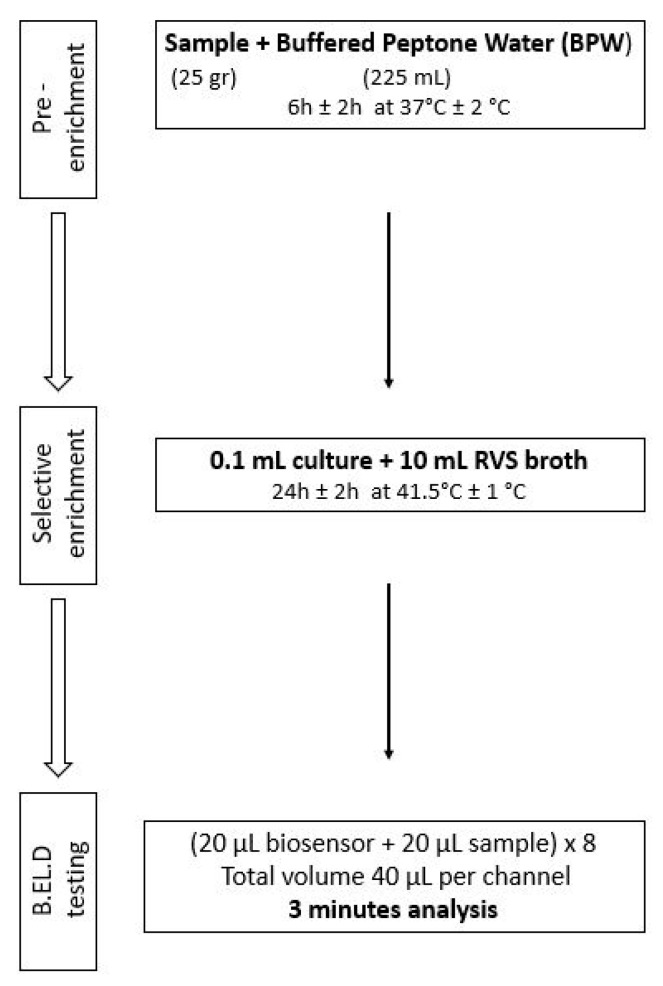
Phase 2—Protocol 5.

**Figure 7:**
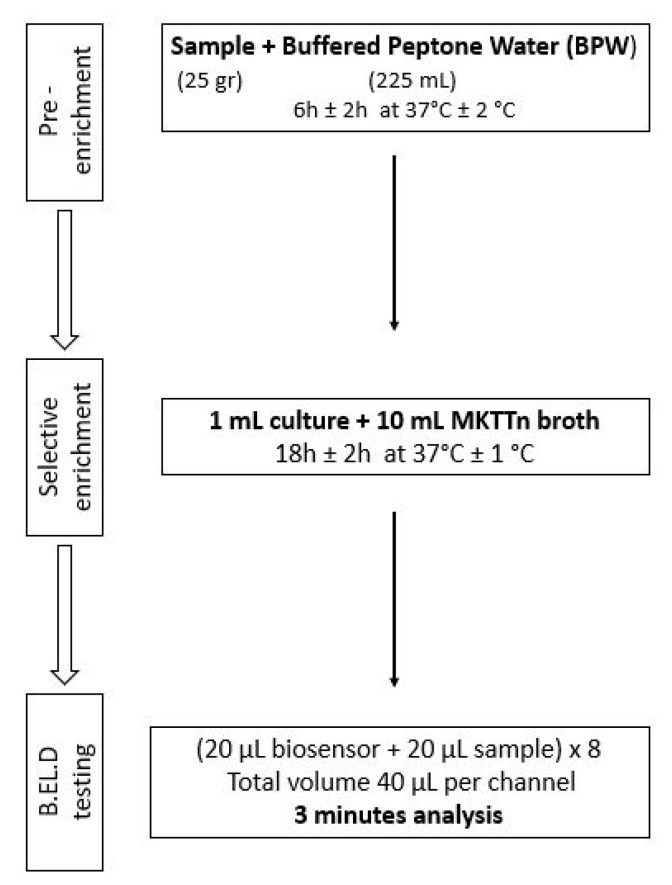
Phase 2—Protocol 6.

**Figure 8:**
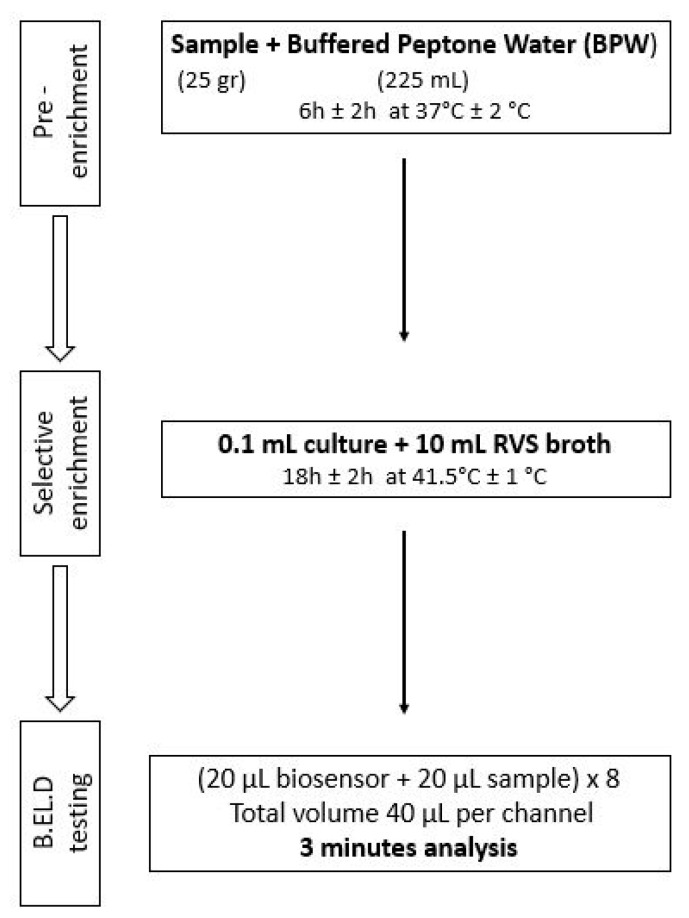
Phase 2—Protocol 7.

As previously mentioned, (Section 2.1), the new method was developed in two phases. During phase 1 three different protocols were followed to evaluate the biosensor’s ability to detect *Salmonella* spp. in the food samples. These protocols (Protocols 1–3) were designed based on the ISO 6579-1:2017 methodology [16] and are presented in Figure 2, Figure 3 and Figure 4. Subsequently, four different protocols were evaluated during phase 2. These protocols (Protocols 4–7) were designed as well based on the ISO 6579-1:2017 [16] methodology, but the incubation periods were significantly reduced to evaluate the biosensor’s ability to detect the pathogen within 30 h or less (Figure 5, Figure 6, Figure 7 and Figure 8).

#### 2.8.1. Protocols

In total, seven protocols with different combinations of nutrient enrichment media and incubation periods were evaluated. All of them are described below in detail.

Protocol 1: Briefly, 25 g of each sample (inoculated or not) were homogenized with 225 mL of BPW in a sterile Stomacher bag. The suspension was incubated for 24 h at 37 °C. After the incubation, 0.1 mL of the suspension was inoculated into 10 mL of Rappaport–Vassiliadis–Soya (RVS) and incubated for 24 h at 41.5 °C. The next day, a portion of the incubated suspension was tested with the biosensor, as described above. In addition to the biosensor analysis, the samples were also examined with the ISO 6579-1:2017 [16] using the selective Xylose Lysine Deoxycholate agar (XLD) for results validation. In the case of uninoculated samples (control samples), the presence of presumptive *Salmonella* spp. colonies, was further investigated through biochemical tests using API20E.

Protocol 2: Briefly, 25 g of each sample (inoculated or not) were homogenized with 225 mL of BPW in a sterile Stomacher bag. The suspension was incubated for 24 h at 37 °C. After the 24-h incubation, 1 mL of the suspension was inoculated into 10 mL of Müller–Kauffmann Tetrathionate (MKTTn) and incubated for 24 h at 37 °C. The next day, a portion of the incubated suspension was tested with the biosensor, as described above. In addition to the biosensor analysis, the samples were also examined with the ISO 6579-1:2017 [16] using the selective XLD agar for results validation. In the case of uninoculated samples (control samples), the presence of presumptive *Salmonella* spp. colonies, was further investigated through biochemical tests using API20E.

Protocol 3: Briefly, 25 g of each sample (inoculated or not) were homogenized with 225 mL of BPW in a sterile Stomacher bag. The suspension was incubated for 24 h at 37 °C. After the incubation, 0.1 mL of the suspension was inoculated into 10 mL of RVS and incubated for 6 h at 41.5 °C. After the 6 h incubation, 1 mL of the RVS suspension was inoculated in 10 mL of M broth and incubated for 24 h at 37 °C. The next day, a portion of the incubated suspension was tested with the biosensor, as described above. In addition to the biosensor analysis, the samples were also examined with the ISO 6579-1:2017 [16] using the selective XLD agar for results validation. In the case of uninoculated samples (control samples), the presence of presumptive *Salmonella* spp. colonies, was further investigated through biochemical tests using API20E.

Protocol 4: Briefly, 25 g of each sample (inoculated or not) were homogenized with 225 mL of BPW in a sterile Stomacher bag. The suspension was incubated for 24 h at 41.5 °C. The next day, a portion of the incubated suspension was tested with the biosensor, as described above. In addition to the biosensor analysis, the samples were also examined with the ISO 6579-1:2017 [16] using the selective XLD agar for results validation. In the case of uninoculated samples (control samples), the presence of presumptive *Salmonella* spp. colonies, was further investigated through biochemical tests using API20E.

Protocol 5: Briefly, 25 g of each sample (inoculated or not) were homogenized with 225 mL of BPW in a sterile Stomacher bag. The suspension was incubated for 6 h at 37 °C. After the 6-h incubation, 0.1 mL of the suspension was inoculated into 10 mL of RVS and incubated for 24 h at 41.5 °C. The next day, a portion of the incubated suspension was tested with the biosensor, as described above. In addition to the biosensor analysis, the samples were also examined with the ISO 6579-1:2017 [16] using the selective XLD agar for results validation. In the case of uninoculated samples (control samples), the presence of presumptive *Salmonella* spp. colonies, was further investigated through biochemical tests using API20E.

Protocol 6: Briefly, 25 g of each sample (inoculated or not) were homogenized with 225 mL of BPW in a sterile Stomacher bag. The suspension was incubated for 6 h at 37 °C. After the 6-h incubation, 1 mL of the suspension was inoculated into 10 mL of Müller-Kauffmann Tetrathionate (MKTTn) and incubated for 24 h at 37 °C. The next day, a portion of the incubated suspension was tested with the biosensor, as described above. In addition to the biosensor analysis, the samples were also examined with the ISO 6579-1:2017 [16] using the selective XLD agar for results validation. In the case of uninoculated samples (control samples), the presence of presumptive *Salmonella* spp. colonies, was further investigated through biochemical tests using API20E.

Protocol 7: Briefly, 25 g of each sample (inoculated or not) were homogenized with 225 mL of BPW in a sterile Stomacher bag. The suspension was incubated for 6 h at 37 °C. After the 6-h incubation, 0.1 mL of the suspension was inoculated into 10 mL of RVS and incubated for 18 h at 41.5 °C. The next day, a portion of the incubated suspension was tested with the biosensor, as described above. In addition to the biosensor analysis, the samples were also examined with the ISO 6579-1:2017 [16] using the selective XLD agar for results validation. In the case of uninoculated samples (control samples), the presence of presumptive *Salmonella* spp. colonies, was further investigated through biochemical tests using API20E.

## 3. Results

**Figure 9:**
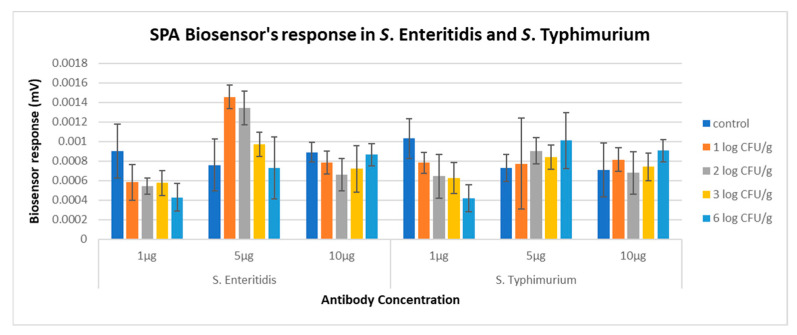
Biosensor response in various antibody SPA concentrations in *S.* Enteritidis and *S.* Typhimurium broth samples.

**Figure 10:**
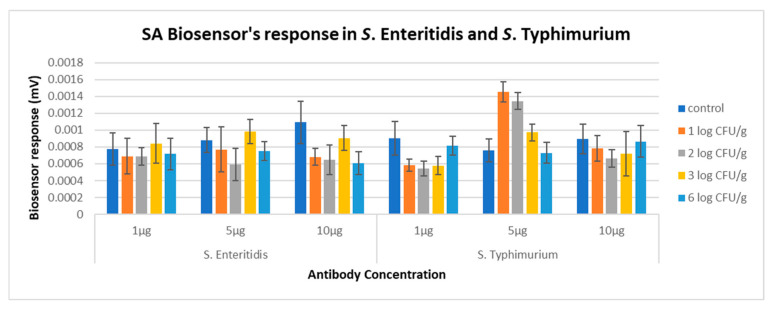
Biosensor response in various antibody SPA concentrations in *S*. Enteritidis and *S.* Typhimurium broth samples.

After analyzing all the data obtained from the experiments contacted according to the description in Section 2.3, results indicated that the best discrimination between samples with and without *Salmonella* spp. was achieved by SPA biosensor with 1 μg mL^−1^ antibody concentration. The biosensor with this antibody at this concentration was able to distinguish blank samples from samples with *Salmonella* Enteritidis and *Salmonella* Typhimurium at all population levels studied, with 84.3% and 82% accuracy, respectively. When the antibody concentration was at 5 and 10 μg mL^−1^, the SPA biosensor was not able to discriminate samples with and without the pathogen, even at high population levels (6 log CFU mL^−1^) (Figure 9). At an antibody concentration of 1 μg mL^−1^, the SPA biosensor demonstrated sensitivities of 82.9% and 85%, specificities of 90% and 70%, and positive predictive values of 97.1% and 91.8% for *Salmonella* Enteritidis and *Salmonella* Typhimurium, respectively. However, its negative predictive values were lower, at 56.2% and 53.8% for the two strains. Additionally, the biosensor exhibited perfect discrimination between samples with the highest inoculum level (6 log CFU mL^−1^) and all other samples, achieving 100% accuracy, sensitivity, specificity, positive predictive value, and negative predictive value for both strains. Contrary to these results, the SA biosensor at 1 μg mL^−1^ antibody concentration could differentiate blank samples from those inoculated with *Salmonella* Typhimurium at any of the concentrations studied with 72% accuracy. However, it was unable to discriminate blank samples from those inoculated with *Salmonella* Enteritidis at any population level (Figure 10). Additionally, the biosensor failed to discriminate between positive and negative samples at 5 or 10 μg mL^−1^ for both serovars. Based on these observations, and its ability to distinguish blank from inoculated samples, particularly when the pathogen was present at high concentrations, the SPA biosensor with 1 μg mL^−1^ antibody concentration was chosen for subsequent testing.

**Figure 11:**
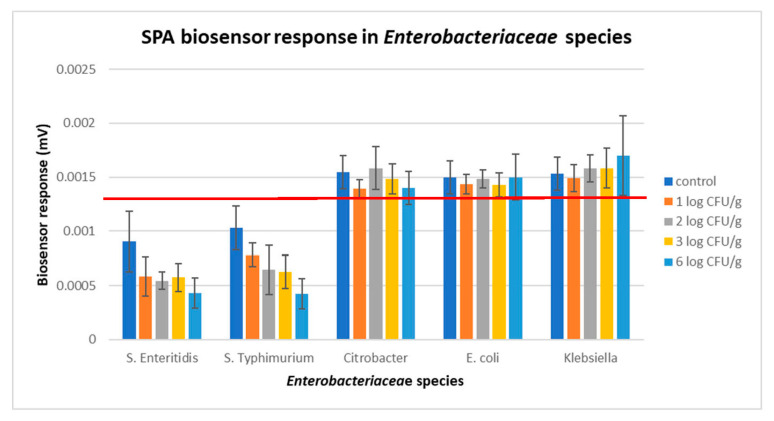
Biosensor response in presence of *Enterobacteriaceae* species. The red line indicates the significant difference in the biosensor response generated in the presence of *Salmonella* spp. compared to other species of the *Enterobacteriaceae* family.

Based on the results obtained from the cross-reactivity assessment (Section 2.4), the biosensor demonstrated a significantly different response between samples containing *Salmonella* spp. and those containing *Citrobacter freundii*, *Escherichia coli*, and *Klebsiella aerogenes* across all tested population levels (Figure 11). Furthermore, in the presence of *Citrobacter freundii*, *Escherichia coli*, and *Klebsiella aerogenes*, the biosensor exhibited the same response across all population levels of these microorganisms, indicating that no reaction occurred when the samples were mixed with the biosensor during analysis. Thus, it was concluded that the newly developed biosensor shows no cross-reactivity with other *Enterobacteriaceae* species.

The results obtained from all seven protocols are summarized in Table 2. As already mentioned, the examined samples were cured meat samples, frozen ready-to-eat meat, and meat preparations, and the study was carried out in two separate phases (Phase 1 and Phase 2). In Phase 1, three different protocols were evaluated for the ability of the biosensor to detect *Salmonella* spp. Subsequently (Phase 2), four different protocols with reduced incubation times were studied to evaluate the biosensor’s ability to detect the pathogen within 30 h or less.

### 3.1. Phase 1

The results obtained from the tests conducted in all three protocols indicated that the biosensor was able to discriminate samples with and without *Salmonella* spp. with high accuracies (83.8–97.7%) when the limit of detection (LOD) was as low as 0.6 log CFU g^−1^. More accurately, the performance characteristics for protocol 1, with an overall incubation time of 48 h, were Acc: 97.7%, Se.: 100%, Sp.: 97%, PPV: 90.9%, and NPV: 100%. For protocol 2, with an estimated incubation time of 48 h, the characteristic indices were Acc: 83.8%, Se.: 66.6%, Sp.: 88%, PPV: 57.1%, and NPV: 91.6%. Finally, for protocol 3, with a 54-h total incubation time, the characteristics were Acc: 90%, Se.: 100%, Sp.: 87.5%, PPV: 66.6%, and NPV: 100%. Hence, it was indicated that protocol 1 had the best discrimination power compared to the other two protocols, revealing the highest accuracy of 97.7%.

**Figure 12:**
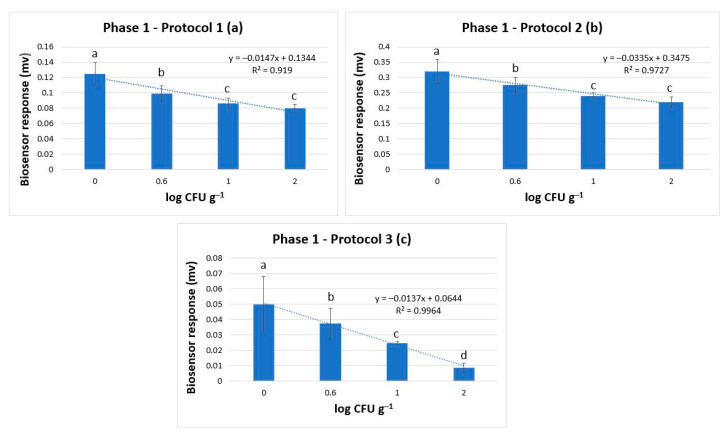
Phase 1: Biosensor response in protocol 1 (**a**), protocol 2 (**b**), and protocol 3 (**c**) in samples without *Salmonella* spp. (0 log CFU g^−1^) and with *Salmonella* spp. at 0.6, 1, and 2 log CFU g^−1^ (initial inoculation level). The error bars represent the standard errors of the mean value of all replications. The columns marked with different letters indicate that the response was significantly (*p* < 0.05) different from the respective one obtained from control samples.

Incubation of the samples with the different enrichment broths augmented *Salmonella’s* population at high levels (≥5 log CFU g^−1^), thus increasing the biosensor’s ability to discriminate positive from negative samples, even when the bacterium was inoculated at very low population levels (0.6 log CFU g^−1^). This was not observed only in protocol 1, but also in protocols 2 and 3 (Figure 12a–c). Furthermore, the results showed that the potential dynamic of the samples (biosensor’s response) was decreasing almost to a linear pattern against increasing *Salmonella* concentrations, with a statistically significant discrimination power (*p* < 0.05) in all three protocols. Hence it was indicated that the newly developed method could detect pathogens in meat and meat products.

### 3.2. Phase 2

The results obtained from the tests conducted in all four protocols of Phase 2 indicated that the biosensor was able to discriminate samples with and without *Salmonella* spp. in less than 30 h. The accuracies, however, were lower than that reported in Phase 1 and fluctuated between 73.3% and 86.1% (Table 1). Furthermore, the LOD increased at 1 log CFU g^−1^. In brief, the performance characteristics of protocol 5, with an estimated incubation time of 30 h, were Acc: 88.8%, Se.: 89.6%, Sp.: 87.5%, PPV: 92.8%, and NPV: 82.3%. The characteristics for protocol 6, with a 24-h total incubation time, were Acc: 78%, Se.: 84.7%, Sp.: 60.7%, PPV: 84.7%, and NPV: 60.7%. Finally, for protocol 7, with a total of 24 h of incubation time, the characteristics were Acc: 86.1%, Se.: 85.7%, Sp.: 86.3%, PPV: 80%, and NPV: 90.5%. Contrary to these results, tests on samples from protocol 4 revealed the method’s lack of ability to produce trustworthy results regarding the pathogen’s absence/presence. More accurately, the performance indices for protocol 4, with an overall incubation time of 24 h, were Acc: 78.5%, Se.: 50%, Sp.: 87.5%, PPV: 55.5%, and NPV: 34.8%. Hence, it was indicated that protocol 7 had the best discrimination power compared to the other four protocols of Phase 2, revealing the highest accuracy of 86.1%.

**Figure 13:**
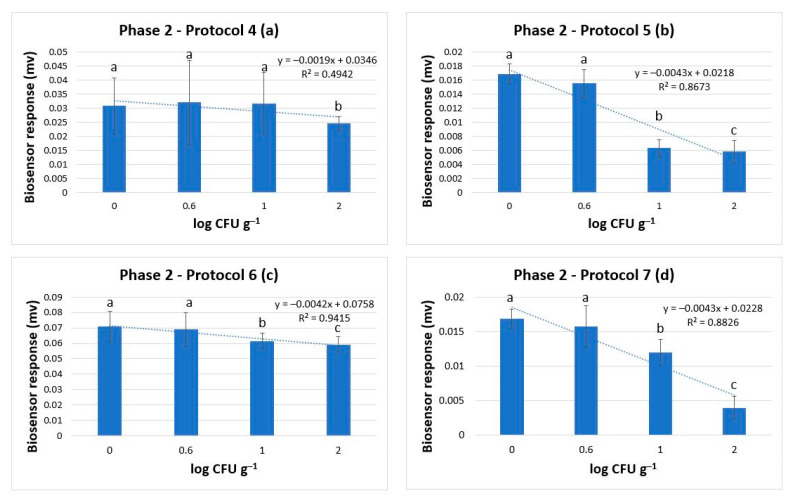
Phase 2: Biosensor response in protocol 4 (**a**), protocol 5 (**b**), and protocol 6 (**c**), and protocol 7 (**d**) in samples without *Salmonella* spp. (0 log CFU g^−1^) and with *Salmonella* spp. at 0.6, 1, and 2 log CFU g^−1^ (initial inoculation level). The error bars represent the standard errors of the mean value of all replications. The columns marked with different letters indicate that the response was significantly (*p* < 0.05) different from the respective one obtained from control samples.

The reduction of the incubation time and the incubation of the samples with the different broths augmented *Salmonella*’s population at levels between 3.5–4 log CFU g^−1^. Thus, the ability of the biosensor to discriminate positive from negative samples decreased compared to the one presented in the first phase of experiments. When the bacterium was inoculated at very low population levels (0.6 log CFU g^−1^), the sensitivity of the biosensor was reduced by approximately 50% (Figure 13). Hence, to increase the accuracy and the performance characteristics of the method, the LOD was set at the higher initial population level of 1 log CFU g^−1^. Of all four protocols (Protocols 4–7), Protocols 5 and 7 were found to have the best discrimination power among the four protocols, with an accuracy of 88.8 and 86.1%, respectively. However, since protocol 7 has the shortest incubation period including 6 h of incubation of the food sample in BPW followed by inoculation of the selective enrichment broth RVS and incubation for 18 h (24 h in total), it was selected as the best protocol for the rapid detection of the pathogen in meat and meat products. Furthermore, it showed an almost linear decrease pattern of the potential dynamic of the samples against increasing *Salmonella* concentration, with statistically significant discrimination power (*p* < 0.05).

## 4. Discussion

In food, *Salmonella* is typically found in low population levels, frequently mixed with much higher concentrations of other microbes. Food processing treatments also can put the pathogen under a lot of stress, notwithstanding its tolerance to harsh conditions. Nevertheless, *Salmonella* can grow to form vast populations and recover its viability and pathogenicity under certain conditions, even if they are initially in minute quantities or in stressed or injured states. It is, therefore, advised to enrich the tested samples to minimize this danger, even for gene-based or immunologically based techniques that can detect the presence of a pathogen in a sample in significantly lower time periods in comparison to the conventional techniques. Hence, enrichment broths were used in every studied protocol, except for Protocol 4.

The first critical step in developing an accurate biosensor system for detecting *Salmonella* spp. was the selection of an appropriate antibody that could respond sensitively and specifically to the presence of the pathogen. The experimental data indicated that the SPA biosensor with an antibody concentration of 1 μg mL^−1^ provides the best discrimination between samples with and without *Salmonella spp.*, showing high accuracy levels of 84.3% for *Salmonella* Enteritidis and 82% for *Salmonella* Typhimurium. This biosensor was able to differentiate blank samples from inoculated ones at all population levels tested. Notably, the biosensor achieved perfect discrimination (100% accuracy, sensitivity, specificity, positive and negative predictive values) for samples with the highest pathogen load (6 log CFU mL^−1^). It was therefore used for subsequent validation tests of the newly developed biosensor system on food.

The validation of the newly developed biosensor system on food was achieved by comparing and evaluating seven different protocols. For the seven protocols evaluated in this study, four different enrichment and pre-enrichment broths (RVS, MKTTn, M broth and BPW) were used with different combinations each time and a different incubation time for each stage of the protocol. BPW is suitable for the preliminary non-selective enrichment of bacteria, especially pathogenic *Enterobacteria* such as *Salmonella* and *Cronobacter*, from food, water, and other materials. It is also rich in nutrients and produces high rates of regeneration for sub-lethal injured bacteria and vigorous growth. The phosphate buffer system prevents bacterial damage caused by changes in the pH of the medium [31]. Both RVS and MKTTn are selective enrichment broths used for the isolation of *Salmonella*. They selectively promote the growth and multiplication of *Salmonella*, while simultaneously inhibiting the growth of other bacteria that happen to be present in the sample [31,32,33]. M broth is a general-purpose enrichment broth used for the cultivation of a variety of microorganisms. M Broth can be used for the detection of *Salmonella*, but it is not as selective as RVS or MKTTn [34].

In the first phase (Protocols 1–3), the biosensor exhibited impressive accuracy in detecting *Salmonella* spp. in meat samples with detection rates ranging from 83.8% to 97.7%, depending on the protocol used. The biosensor’s ability to detect the pathogen was enhanced by incubating the samples with different enrichment broths, which increased *Salmonella*’s population levels, even when the bacterium was inoculated at very low levels (0.6 log CFU g^−1^). In the second phase (Protocols 4–7), successfully discriminated between *Salmonella*-positive and -negative samples within a shorter timeframe—less than 30 h for Protocols 5, 6, and 7. However, the accuracy was lower than in the first phase, ranging from 73.3% to 88.8%, and the LOD increased to 1 log CFU g^−1^. This reduction in performance was linked to shorter incubation times and the absence of specific enrichment steps, leading to decreased accuracy and sensitivity. Notably, in tests using Rappaport-Vassiliadis Soya (RVS) broth as the main enrichment medium (Protocols 1, 5, and 7), the method’s accuracy decreased from 97.7% to 88.6% and 86.1% when the total incubation period was reduced from 48 h to 30 h and 24 h, respectively. Likewise, when MKTTn was the main enrichment broth (Protocols 2 and 6), accuracy dropped from 83.8% to 78% with a reduction in incubation time from 48 h to 24 h. The protocols in Phase 1 allowed the pathogen population to grow to higher levels (≥5 log CFU g^−1^) compared to those in Phase 2 (3.5–4 log CFU g^−1^), which explains the anticipated drop in accuracy. As the population of *Salmonella* increased with longer incubation, the biosensor’s response became more pronounced, highlighting the importance of sufficient incubation for optimal performance. In general, the protocols utilizing the RVS broth presented higher discrimination power than the protocols where MKTTn broth was used. The higher sensitivity of RVS compared to other *Salmonella* selective enrichment media, especially when utilizing low inoculation levels of pre-enrichment broth, has been also reported by Vassiliadis in 1983 [35].

Protocol 3, which involved the longest incubation period, demonstrated high accuracy (90%) and strong performance characteristics. However, it was ultimately rejected due to the lengthy procedure time. In contrast, Protocol 4, with the shortest incubation time, proved unsuitable because it lacked the ability to effectively discriminate *Salmonella* spp., even when the pathogen was initially inoculated at high population levels (2 log CFU g^−1^). This limitation was attributed to the fact that BPW, that was the only broth used for sample preparation in this protocol, is a non-selective pre-enrichment broth that promotes the growth of a variety of microorganisms. Consequently, the absence of selective inhibitors allowed other bacteria in the sample, potentially present in much higher numbers, to flourish, further hindering *Salmonella* detection. Additionally, results from Protocol 4 indicated that the potential dynamics measured in samples with and without *Salmonella* were remarkably similar, especially at initial inoculation levels (0.6 and 1 log CFU g−^1^). Since the membrane potential of microorganisms, which is critical for their behavior, is not homeostatic [36], the observed potential dynamics were not solely attributed to the antigen-antibody reaction but also to changes in bacterial membrane potential, which occur for survival. A statistically significant decrease in potential dynamic was only observed when the pathogen was inoculated at 2 log CFU g^−1^, yet even at this level, the method failed to achieve reliable discrimination. Notably, despite differences between control and highly inoculated samples, the method could not distinguish negative from positive results without the addition of the biosensor. This finding underscored the critical role of the antibody-antigen reaction in the newly developed system, demonstrating that the biosensor was correctly designed for its purpose. In summary of the above, Protocol 7 was selected as the best protocol for the rapid detection of the pathogen in meat and meat products. This protocol had the shortest incubation period (24 h in total) and revealed robust performance characteristics ranging from 80 to 90.5% while the limit of detection was determined to be as low as 1 log CFU g^−1^ in all food substrates.

In recent years, *Salmonella* rapid detection methods have advanced significantly due to the high risk posed by food contamination. Various bioreceptors have been explored for detecting *Salmonella*, and while these innovations have improved accuracy to approach that of traditional culture and gene sequencing methods, each technique still presents limitations. For example, colorimetry, though simple and effective for preliminary identification, can be compromised by the color of the sample. Fluorescence methods offer high sensitivity but are affected by background noise from the sample matrix. While the Surface-Enhanced Raman Spectroscopy (SERS) shows promise due to its strong resistance to interference, it requires more precise, miniaturized instruments to reach its full potential. Surface Plasmon Resonance (SPR) is capable of real-time monitoring but suffers from low sensitivity, requiring further optimization. Photothermal detection introduces new possibilities but remains hindered by instability and low sensitivity. Finally, existing electrochemical biosensors, despite their ultra-high sensitivity, struggle to perform in the presence of complex food matrices, necessitating extensive sample pretreatment to ensure reliable results [37]. These drawbacks highlight the need for ongoing improvements to further enhance the reliability and applicability of rapid *Salmonella* detection in diverse food environments.

**Figure 14:**
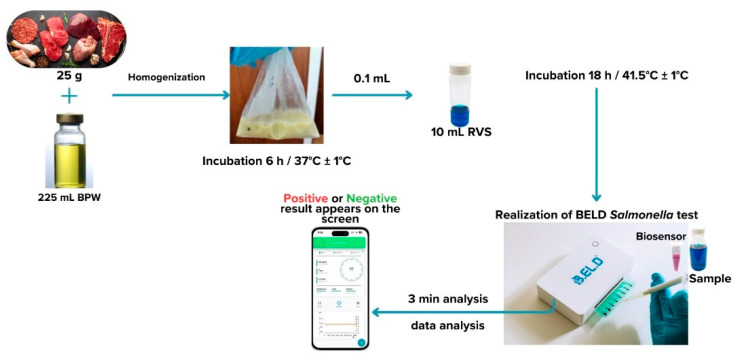
Schematic diagram of the newly developed *Salmonella* spp. detection process.

Biosensors are widely regarded as one of the most effective systems for detecting *Salmonella*, with broad applications across multiple fields, including medical diagnostics, food safety, environmental monitoring, drug delivery, and beyond [38]. Moreover, recent reviews emphasize the need for ongoing advancements in automated detection technologies, in conjunction with big data analytics and artificial intelligence (AI), to enhance the efficacy and precision of food safety monitoring. It is also highlighted that ideal rapid detection systems for *Salmonella* should minimize manual steps, be cost-effective to manufacture, and user-friendly. Furthermore, with smartphones already playing a role in rapid detection, miniaturization and automation of detection devices have emerged as key trends for developing more competitive strategies [37,38]. The newly developed method incorporates these characteristics—a biosensor technique utilizing a portable, user-friendly device integrated with big data analytics and AI technology—representing a significant advancement in *Salmonella* detection (Figure 14). Therefore, there are various application aeras, including routine testing in food processing facilities, rapid screening in import/export control, and on-site testing in food safety inspection where this technology could be very useful. Finally, since this technology is an ideal tool for rapid *Salmonella* detection, future research could explore adaptation for detecting other foodborne pathogens and enhancing the biosensor’s sensitivity and specificity for diverse sample types.

## 5. Conclusions

This study has demonstrated the effectiveness of a novel, portable cell-based biosensor system for the detection of *Salmonella enterica* ser. Enteritidis and *Salmonella enterica* ser. Typhimurium in meat and meat products. Developed in alignment with the ISO 6579-1:2017 standard [16], the biosensor is designed to seamlessly integrate into routine lab diagnostics, offering both precision and convenience. With just a 24-h enrichment step and a rapid 3-min analysis, the system provides reliable results in a fraction of the time required by traditional methods.

This biosensor stands out for its user-centric design, integrating seamlessly with a mobile app via Bluetooth 4.0 for Android and iOS devices, providing real-time access to food safety test results. All data are securely stored in the cloud, accessible through an API, and managed in a secure database, enabling users to track their test history. By leveraging machine learning algorithms, big data analytics, and artificial intelligence (AI), the system enhances the accuracy and efficiency of food safety monitoring, providing a powerful tool for ensuring food quality and safety.

With an accuracy of 86.1% and a limit of detection (LOD) of 1 log CFU g^−1^, this innovative technology enables fast and sensitive identification of *Salmonella* spp. in meat and meat products, making it an ideal screening tool for the rapid detection of the pathogen. The combination of speed, accuracy, and modern connectivity makes this biosensor a game-changing solution for food safety, providing a powerful, practical tool for ensuring the safety of meat products, while offering scalability for broader commercial applications.

## Figures and Tables

**Table 1:** Overview of recent methods for detecting *Salmonella* in food products, highlighting their advantages and disadvantages.

Method	Description	Advantages	Disadvantages
Culture-based methods	Traditional—gold standard method	High accuracy and specificity; isolating live bacteria	Time-consuming (5–7 days); labor-intensive; requires skilled personnel
Polymerase Chain Reaction (PCR) Real-Time PCR (qPCR)	DNA-based detection of *Salmonella*-specific genes	High sensitivity and specificity; fast; widely validated and automated options	False negative result if target gene is absent or mutated; complex procedures; requires skilled personnel; high cost of equipment and consumables
Immunoassay-based methods	Detection based on *Salmonella* antigen-antibody interactions.	Relatively quick; suitable for routine screening	Lower sensitivity; false positive results due to cross-reactivity with similar antigens
Whole-Genome Sequencing (WGS)	Identification of *Salmonella* strains	Provides detailed genetic information; valuable for outbreak investigations and tracing	Expensive and complex; requires bioinformatics expertise; long processing time
Biosensors	*Salmonella* detection via electrochemical, optical, or piezoelectric signals	Real-time detection; rapid; can be highly sensitive and specific; potential for field testing	Still emerging; method often requires optimization; cost and complexity vary by type

**Table 2:** Performance indices of the seven protocols studied.

Performance Indices	Protocol 1	Protocol 2	Protocol 3	Protocol 4	Protocol 5	Protocol 6	Protocol 7
	RVS 48 h	MKTTn 48 h	M broth 54 h	BPW 24 h	RVS 30 h	MKTTn 24 h	RVS 24 h
Accuracy	97.7%	83.8%	90%	78.5%	88.8%	78%	86.1%
Se. ^a^	100%	66.6%	100%	50%	89.6%	84.7%	85.7%
Sp. ^b^	97%	88%	87.5%	87.5%	87.5%	60.7%	86.3%
PPV ^c^	90.9%	57.1%	66.6%	55.5%	92.8%	84.7%	80%
NPV ^d^	100%	91.6%	100%	34.8%	82.3%	60.7%	90.5%

^a^ Se.: Sensitivity; ^b^ Sp.: Specificity; ^c^ PPV: Positive Predictive Value; ^d^ NPV: Negative Predictive Value.

## Data Availability

Data is contained within the article.
